# Automated search methods for identifying wrong patient order entry—a scoping review

**DOI:** 10.1093/jamiaopen/ooad057

**Published:** 2023-08-02

**Authors:** Mathew Garrod, Andy Fox, Paul Rutter

**Affiliations:** Department of Pharmacy, University Hospital Southampton NHS Foundation Trust, Southampton, UK; Department of Pharmacy, University Hospital Southampton NHS Foundation Trust, Southampton, UK; School of Pharmacy and Biomedical Science, University of Portsmouth, Portsmouth, UK

**Keywords:** computerized provider order entry, electronic prescribing, medication errors, surveillance, error detection

## Abstract

**Objective:**

To investigate: (1) what automated search methods are used to identify wrong-patient order entry (WPOE), (2) what data are being captured and how they are being used, (3) the causes of WPOE, and (4) how providers identify their own errors.

**Materials and Methods:**

A systematic scoping review of the empirical literature was performed using the databases CINAHL, Embase, and MEDLINE, covering the period from database inception until 2021. Search terms were related to the use of automated searches for WPOE when using an electronic prescribing system. Data were extracted and thematic analysis was performed to identify patterns or themes within the data.

**Results:**

Fifteen papers were included in the review. Several automated search methods were identified, with the retract-and-reorder (RAR) method and the Void Alert Tool (VAT) the most prevalent. Included studies used automated search methods to identify background error rates in isolation, or in the context of an intervention. Risk factors for WPOE were identified, with technological factors and interruptions deemed the biggest risks. Minimal data on how providers identify their own errors were identified.

**Discussion:**

RAR is the most widely used method to identify WPOE, with a good positive predictive value (PPV) of 76.2%. However, it will not currently identify other error types. The VAT is nonspecific for WPOE, with a mean PPV of 78%–93.1%, but the voiding reason accuracy varies considerably.

**Conclusion:**

Automated search methods are powerful tools to identify WPOE that would otherwise go unnoticed. Further research is required around self-identification of errors.

## BACKGROUND AND SIGNIFICANCE

A medication error (ME) is a failure in the treatment process that leads to, or has the potential to lead to, harm to the patient.^1^ The US Food and Drug Administration (FDA) receives more than 100 000 reports each year associated with suspected MEs,^2^ however the true number is thought to be much higher. Studies have estimated that in the United States preventable MEs affect more than 7 million patients per year, contributing to 7000–9000 deaths, and costing almost $21 billion.^3^^,^^4^ In England, it is estimated that MEs cost the National Health Service (NHS) over £98 million annually, consuming 181 626 bed-days, and causing or contributing to over 1700 deaths.^5^

Research published over 2 decades ago reported general prescribing error rates of 0.4%–15.4% from the United States^6^^,^^7^ and 7.4%–18.7% from the United Kingdom.^8^^,^^9^ More recent studies from England and Scotland have reported rates of 8.9% and 7.5%, respectively.^10^^,^^11^

Electronic health records (EHR) and electronic prescribing using computerized provider order entry (CPOE) systems have been touted as tools able to prevent and mitigate potential MEs,^12^^,^^13^ thereby improving patient safety.^14^^,^^15^ However, the use of CPOE is not without its problems. Several areas of concern have arisen around the unintended consequences of CPOE usage, which includes more/new work for clinicians and unfavorable workflows, and has been reported as not reliably preventing patient harm in some hospitals.^16^

In addition, the generation of new kinds of errors such as juxtaposition errors, where users select the incorrect option from dropdown lists, has been reported.^17–19^ This has increased the risk of one specific type of error; wrong-patient selection.^19–21^ Although studies indicate that practitioners place more than 99.9% of all orders for the correct patient,^22–25^ the sheer volume of orders placed by practitioners in the United States suggests that an error rate of less than 1 in 1000 would result in approximately 600 000 wrong-patient order entry (WPOE) errors each year.^25^ For an UK teaching hospital placing 2.5 million prescribing orders annually, this would equate to approximately 2500 WPOE errors per year.

As stated by Abraham et al,^26^ while significant research on CPOE-related MEs does exist,^27^^,^^28^ the errors that occur during the medication ordering stage have not been explored thoroughly.^19^^,^^29^ As a result, the current understanding of medication ordering errors is mainly based on retrospective data collected from less robust sources.^30–37^

The value of recording and tracking MEs has been repeatedly highlighted,^38^^,^^39^ with medication safety experts characterizing the current state of unsafe medication ordering practices to be “still a work in progress.”^40^ To address this, recent initiatives have focused on ways to identify and classify medication ordering errors,^37^ however, there are limited, if any, approaches for automatically flagging, tracking, and aggregating medication ordering error data in real time.^41^

One approach to identify near-miss WPOE currently being used has been termed the “retract-and-reorder” (RAR) method. This method specifically assesses the sequence of order placement, retraction within 10 minutes, and subsequent re-entry on a different patient by the same practitioner within a further 10 minutes in order to identify possible wrong-patient selection.^22^ Endorsed by the National Quality Forum for assessing WPOE,^42^ the RAR method has since been used in a number of observational studies and randomized trials.^22–25^^,^^43–46^

The authors have not identified any other reviews assessing WPOE. To our knowledge this is the first such review of its type.

## OBJECTIVES

In this scoping review we aimed to explore the literature related to automated error detection for WPOE. This review was conducted to comply with the PRISMA-ScR statement for scoping reviews. The objectives of this review were to investigate:

1) What automated search methods are being used to identify WPOE?
2) What data are being captured by the automated methods? What are they being used for?
3) What are the causes of WPOE?
4) How are the providers identifying their own errors?

## MATERIALS AND METHODS

### Search strategy

The published empirical literature, from inception up until November 2021, was searched for papers related to the use of automated searching for WPOE when using a CPOE system.

An “automated” search method was defined as one that could return the required data with minimal human input, such as an automatically generated alert from a continuously running search program, a report that is instigated manually, or a report that could be scheduled to run automatically at regular intervals.

The databases searched were *CINAHL, Embase (<1974 to 2021 November 16>), and MEDLINE (<1946 to November 16, 2021>).*

Search terms were primarily related to electronic prescribing and errors. The search terms were extremely broad by necessity as the keywords used in previously identified relevant literature were very generic (eg, “patient safety”). More specifically, the first group of search terms relates to the various different terms for electronic prescribing, the second group relates to types of errors and patient safety, and the third group relates to automated detection and the previously identified RAR process. Titles and abstracts were searched as well as subject headings.

Terms within groups were combined using the Boolean operator “OR” and sets were combined with the Boolean operator “AND” to obtain the final search pool. The search strategy can be found in Supplementary Appendix S1.

### Eligibility criteria

All study designs that focused on the automated detection of WPOE in a CPOE system were included. Studies were excluded if they did not feature both the use of a CPOE system, and an automated search method used to identify WPOE.

English language restrictions were applied and the papers had to have been peer-reviewed.

### Screening

Following the search, all identified citations were uploaded to the web tool Rayyan^®^ (Rayyan Systems Inc, Abu Dhabi, UAE) to facilitate the screening process. After the removal of duplicate titles, abstracts were screened independently by 2 reviewers (MG and PR) and where conflicts arose, these were resolved through retrieval of full-text articles. If unresolved, a third reviewer (AF) was included and a consensus reached.

### Data extraction and synthesis

One researcher (MG) independently undertook data abstraction from each study using a customized data extraction form, with data then reviewed by the remaining researchers (AF, PR). Variables included: author and year; title of the study; country of origin; aims and objectives; setting/specialty; sample size; details of participants; study design; duration; automatic monitoring method; CPOE system; results (qualitative and quantitative); key findings; limitations and further studies.

### Definitions for extracted items

A “voided” order is an order that was discontinued by a clinician. The “void” method of discontinuation is used to indicate the order was placed in error.

WPOE was defined as placing any order (eg, medication, lab request, etc.) on the wrong patient, rather than the appropriate one.

### Synthesis of results

Thematic analysis was performed using the themes identified by Abraham et al^26^ These were developed using a deductive coding and agreement approach and based on the Systems Engineering Initiative for Patient Safety (SEIPS) risk factors; technological, cognitive, social, environmental, and organizational.^47^ These risk factors contribute to ordering errors in general and are therefore relevant to WPOE. See Supplementary Appendix S2 for more detail.

## RESULTS

### Study characteristics

**Figure 1.**
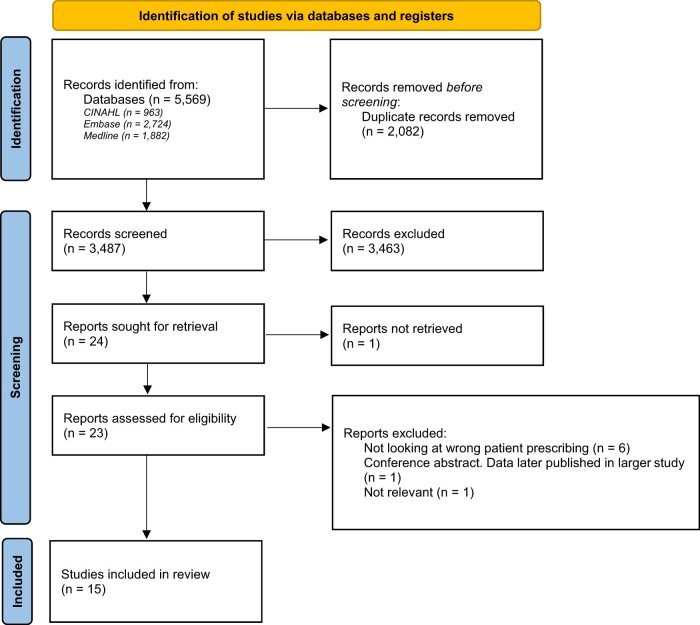
PRISMA chart of study selection process.

The titles and abstracts of 3487 papers, and the full texts of 23 papers, were screened. Fifteen papers were identified as meeting the inclusion criteria. Figure 1 illustrates the study selection and exclusion/inclusion processes.

All papers were US-based, with 10 conducted in a single site and 5 over multiple sites. Eight employed an interventional design type and 7 were noninterventional.

The papers used a variety of different study designs and methodologies, 6 were retrospective,^45^^,^^48–52^ 4 prospective,^26^^,^^41^^,^^46^^,^^53^ and 5 ambidirectional.^22^^,^^23^^,^^43^^,^^54^^,^^55^ Almost half the studies (*n* = 7) included a qualitative component. Study durations ranged from 24 days to 6 years.

A range of commercial and proprietary EHR systems was used in the studies, with some studies using different systems across different sites.

Five papers described search functions utilizing the reason for discontinuation entered by the provider to identify WPOE. Nine papers described the RAR identification method of Adelman et al.^22^

The error rates were originally reported in 4 different ways; per 100 000 orders (*n* = 5), per 1000 orders (*n* = 4), % (*n* = 5), or “events” per week (*n* = 1) (see Table 2 for more details).

### Objective 1: automated search methods found in the literature used to identify WPOE (brief summary)

Table 1 below briefly summarizes the automated search methods found in the literature and groups them together for comparative purposes.

**Table 1.** Automated search tool summary table

Identifies all errors		
Method type	Designated name of search method	Method details
Rapidly discontinued medication^46^	*No name assigned*	Medication orders discontinued within 1 min to 2 h
Interrogation of reasons entered of when a provider voids/discontinues an order^41^^,^^50^^,^^55^	Void Alert Tool (VAT)	Medication orders discontinued with “void” as the reason
		Medication orders discontinued with “void” as the reason for inpatients >18 years old
		Medication orders discontinued with “error (erroneous entry)” as the reason

Identifies WPOE specifically		
Method type	Designated name of search method	Method details
Retract-and-reorder^22^^,^^48^	*No name assigned*	Order retracted within 120 min, then reordered on a different patient within 5 min of retraction
	“Retract-and-reorder”	Order retracted within 10 min, then reordered on a different patient within 10 min of retraction.
Indication-based alert coupled with retract-and-reorder^49^	*No name assigned*	Pre-existing CDS alert triggered if medication ordered did not correlate to an active indication in the patient’s medical record. Alerts for orders started but not completed were analyzed using RAR method of Adelman et al^22^

Abbreviations: CDS: clinical decision support; RAR: retract-and-reorder.

### Objective 1: automated search methods found in the literature used to identify WPOE (detailed)

Table 2 summarizes the parameters of the search methods used in the papers. Where calculated, the positive predictive value (PPV) details how accurate the results from the search are.

**Table 2.** Details of automated search methods found in the literature

Automated search methods identifying all errors					
Papers	Research goal of article	Nature of the search	Voiding parameters	PPV for medication errors	Further relevant information
Koppel et al^46^	Investigating if rapid discontinuations of prescription orders indicate prescribing errors	Prospective, real-time	Medication orders discontinued within 1 min to 2 h	66% (95% CI, 53%–77%) for orders stopped within 45 min being deemed inappropriate. 55% (95% CI, 46%–64%) for orders stopped within 2hrs being deemed inappropriate	
Kannampallil et al^50^	Exploring if order voiding function identifies medication ordering errors	Retrospective	Pulled all the data on medication orders and focused on “voided” orders	70 ± 10% for medication ordering errors 48 ± 10% for accuracy of “wrong patient” voiding reason	
Abraham et al^41^	Investigating accuracy of voiding function in medication ordering errors, and their root causes	Prospective, real-time	Alert generated when “voiding” action performed on an inpatient >18 years old	93.1% (95% CI 88.1%–98.1%) for medication ordering errors 70.2% (95% CI 61.0%–79.4%) for accuracy of voiding reason	Search method termed Void Alert Tool (VAT). Inspired by Kannampallil et al^50^ paper
Hickman et al^55^	Elucidating reasons for discontinuing self-identified erroneous medication orders	Ambidirectional—retrospective data collection. Subsequent prospective study using near-real-time (within 24 h) using automated alert	All medication discontinued the previous day due to “error (erroneous entry)”	*Not reported*	
Abraham et al^26^	Identifying medication ordering error types, investigating their risk factors and mitigation strategies	Prospective, real-time	VAT of Abraham et al^41^	78 ± 1.2% for medication errors 80.3 ± 1.5% for accuracy of voiding reason	PPVs slightly different compared to their previous study

Automated search methods identifying WPOE specifically					
Papers	Research goal of article	Nature of the search	Voiding parameters	Positive predictive value for medication WPOE	Further relevant information
Levin et al^48^	Establishing risk factors for WPOE	Retrospective	Medication cancelled within 2 h of ordering; same drug then reordered on different patient within 5 min of cancellation	*Not reported*	
Adelman et al^22^	Assessing the effectiveness of the RAR method and using it to estimate the frequency of WPOE	Ambidirectional. Retrospective search to develop RAR method, then prospective use during RCT	Order retracted within 10 min, then reordered by the same provider on a different patient within 10 min of retraction.	76.2% (95% CI 70.6%–81.9%)	Search method termed Retract-and-Reorder (RAR)
Galanter et al^49^	Determining whether indication-based CPOE alerts intercept WPOE	Retrospective	Pre-existing clinical decision support (CDS) alert triggered if medication ordered did not correlate to an active indication in the patient’s medical record. Alerts then analyzed using RAR method of Adelman et al^22^	*Not reported*	
Green et al^23^	Evaluating the impact of a CPOE-based patient verification intervention to reduce WPOE	Ambidirectional—gathered WPOE rate pre- and postintervention	RAR method of Adelman et al^22^	76.2% (95% CI 70.6%–81.9%)	Study quotes PPV from Adelman et al^22^
Adelman et al^43^	Evaluating if assigning distinct first names at birth reduces WPOE	Ambidirectional—gathered WPOE rate pre- and postintervention	RAR method of Adelman et al^22^	76.2% (95% CI 70.6%–81.9%)	Study quotes PPV from Adelman et al^22^
Lombardi et al^54^	Evaluating the impact of a CPOE-based patient verification intervention to reduce WPOE	Ambidirectional—gathered WPOE rate pre- and postintervention	RAR method of Adelman et al^22^	76.2% (95% CI 70.6%–81.9%)	Study quotes PPV from Adelman et al^22^
Adelman et al^45^	Assessing the risk of WPOE in multiple-birth infants vs singletons in neonatal intensive care	Retrospective	RAR method Adelman et al^22^	*Not reported*	
Salmasian et al^53^	Evaluating if patient photos reduce WPOE	Prospective	RAR method of Adelman et al^22^	76.2%	Study quotes PPV from Adelman et al^22^
Kern-Goldberger et al^51^	Comparing WPOE rates on obstetric units in different patient groups	Retrospective	RAR method of Adelman et al^22^	*Not reported*	
Udeh et al^52^	Assessing if reducing number of concurrently open EHRs reduces WPOE	Retrospective	RAR method of Adelman et al^22^	*Not reported*	

Abbreviations: PPV: positive predictive value; WPOE: wrong-patient order entry; CI: confidence interval; RAR: retract-and-reorder; RCT: randomized controlled trial; VAT: void alert tool.

### Objective 2: error data captured by the automated methods and how the methods are being used

Table 3 below displays the error data being captured by the automated search method. It also details if the method was used to assess the success of an intervention, or to gather background error data. For ease of comparison, the papers are grouped together based on the method used.

**Table 3.** Error data captured by the automated methods and how it is being used

Automated search methods identifying all errors				
Paper	Descriptive information about error timings	Background error rate	Intervention error rate	Further relevant information
*Used unique method of Koppel et al^46^*				
Koppel et al^46^	Orders stopped within 30 min were most likely to be deemed inappropriate	*Not specified*	*No intervention*	
*Method utilizing the descriptive discontinuation option native to the CPOE system (eg, “void,” “Error—erroneous entry”)*				
Kannampallil et al^54^	*Not specified*	490 “voids” per 100 000 orders (0.49%)	*No intervention*	Originally reported as per 1000 orders
Abraham et al^41^	*Not specified*	210 “voids” per 100 000 orders (0.21%)	*No intervention*	Originally reported as per 1000 orders
Hickman et al^55^	*Not specified*	450 “errors” per 100 000 orders (0.45%)	*No intervention*	Originally reported as per 1000 orders
Abraham et al^26^	Median time from medication ordering to its voiding was 0.38 h	*Not specified*	*No intervention*	Primarily a qualitative paper

Automated search methods identifying WPOE specifically				
Paper	Descriptive information about error timings	Background error rate	Intervention error rate	Further relevant information
*Used unique method of Levin et al^48^*				
Levin et al^48^	Median retract time (1 min) and reorder time (1 min) Highest risk of WPOE on Fridays, and 12:01 am to 6 am	64 per 100 000	*No intervention*	Originally reported as 0.064%
*Used RAR method of Adelman et al^22^*				
Adelman et al^22^	Mean time to retraction of 1 min and 18 s	58 per 100 000 orders	1.5 per 1000 order sessions (control) 1.2 per 1000 sessions (intervention 1) 0.9 per 1000 sessions (intervention 2) (RAR rates)	WPOE calculated by dividing RAR events by PPV of RAR method by study authors. Ordering session—when providers selected a patient and then placed one or more orders.
Green et al^23^	*Not specified*	202 per 100 000 orders	4 months later—141 per 100 000 orders 2 years later—153 per 100 000 orders	Originally reported as per 1000 orders Intervention was a patient ID-verify alert
Adelman et al^43^	*Not specified*	45 per 100 000 orders	29 per 100 000 orders	WPOE calculated by dividing RAR events by PPV of RAR method by review team
Lombardi et al^54^	*Not specified*	6.125 “events” per week	4 “events” per week	Intervention was a patient ID-reentry function
Adelman et al^51^	*Not specified*	31.8 per 100 000 orders (single births) 50.3 per 100 000 orders (multiple births)	*No intervention*	WPOE calculated by dividing RAR events by PPV of RAR method by review team
Salmasian et al^53^	*Not specified*	142 per 100 000 orders	101 per 100 000 orders	WPOE calculated by dividing RAR events by PPV of RAR method by review team Intervention was displaying patient photographs within the EHR
Kern-Goldberger et al^51^	*Not specified*	60.6 per 100 000 order sessions (obstetrics) 32.2 per 100 000 order sessions (medical-surgical)	*No intervention*	WPOE calculated by dividing RAR events by PPV of RAR method by review team
Udeh et al^52^	*Not specified*	27.8 per 100 000 orders	21.1 per 100 000 orders	WPOE calculated by dividing RAR events by PPV of RAR method by review team
*Unique method of Galanter et al^49^ coupled with RAR method of Adelman et al^22^*				
Galanter et al^49^	*Not specified*	*Not specified*	25 per 100 000 alerts	Originally reported as per 1000 orders Intervention is an indication-based CDS alert

Abbreviations: WPOE: wrong-patient order entry; RAR: retract-and-reorder; PPV: positive predictive value.

### Objective 3: risk factors for WPOE

Of the 15 papers included in this review, only 7 had qualitative components where the providers were interviewed.^22^^,^^26^^,^^41^^,^^46^^,^^48^^,^^54^^,^^55^ Across these 7 papers the volume of qualitative data varied considerably. Most studies used the automated triggers to contact providers shortly after an event occurred, which is excellent for data collection as the errors should be fresh in the mind of the interviewees. However, Levin et al^48^ obtained a lot of their qualitative data from a local and national survey that was done in addition to their automated search method work. As a result, some of the root causes they identified could be preconceived opinions from the survey respondents and not real-world data. Finally, Abraham et al^26^ had the largest qualitative component by far, reporting extensively on their almost 400 interviews and surveys with almost 300 clinicians.

#### Technological risk factors

After interviewing almost 300 clinicians, the data obtained by Abraham et al^26^ reported that technological factors were the primary contributors to wrong-encounter errors.

One study has reported that only 10% of wrong-patient orders are classified as juxtaposition errors.^22^

Another cause of WPOE may be the EHR feature that allows users to view multiple charts on one screen simultaneously,^48^ although this is disputed. Clinicians are able to have multiple charts open on one computer and toggle back and forth between patients, potentially completing an order on the wrong-patient.^41^^,^^54^ This feature is frequently used in Emergency Departments in the United States, and is believed to be one of the reasons that the rate of patient misidentification is higher in this setting compared to inpatient settings.^48^ However, other studies have demonstrated that having multiple open records does not increase the incidence of WPOE.^24^^,^^25^

Issues with the usability of CPOE systems have been identified. Slow speeds during order verification and modification can give rise to user frustration and inattentive clicking. The large number of steps required to click through, described as “clicking barriers”, has also given rise to clicking fatigue.^26^

Other reported causes include information being displayed differently in the CPOE user interface views between physicians and nurses,^26^ CPOE alert notifications being indirect and lengthy resulting in alert fatigue,^26^^,^^54^ and inadequate CPOE training.^26^

#### Cognitive risk factors

Abraham et al^26^ also showed that cognitive factors predominantly contributed to errors relating to wrong patient.

Similarities between patient age, surname, bed proximity, medical service, time/date of order, ordering intensity, and medication name have all be shown to increase the risk of WPOE.^48^^,^^55^ However, there is conflicting evidence whether patients with similar conditions increase the risk of WPOE.^26^^,^^48^

Physicians have stated that they believe lack of sleep and/or lack of experience are risk factors for WPOE. However, results from one case-control study do not demonstrate fatigue contributing to patient misidentification.^48^

#### Environmental risk factors

The most commonly reported environmental risk factors from the interviews and surveys conducted by Abraham et al^26^ were interruptions, distractions, noise, and time constraints.

One study reports that over 80% of wrong-patient orders are due to interruption events.^22^ This is also corroborated by other studies, with clinicians reporting frequent interruptions from nurses and other physicians while they are trying to place orders.^26^^,^^48^^,^^54^ One clinician interviewed stated that nurses and other physicians take advantage of the fact that they are sitting down at a computer in order to ask them questions.^48^

Some clinicians report feeling considerable time pressure, which resulted in rushing, lack of clinical reviews, and not communicating unique orders effectively.^26^ Rather worryingly, one clinician described a practice of relying on the pharmacist to catch any errors caused by placing orders in a hurry and without due care.^26^

#### Organizational risk factors

The biggest organizational risk factor for errors reported by clinicians in Abraham et al^26^ was their workload. They report not having enough time to review medications appropriately before ordering them, and that their heavy workload results in stress. “*Too many orders*” and “*Multiple order entries*” were also identified as contributing factors for WPOE by Lombardi et al.^54^

Lack of staffing has been reported, which leads to clinicians looking after unfamiliar patients with limited knowledge about them and their clinical picture. A lack of senior staff has also been reported, which resulted in an error caused by a more junior member of staff who needed help.^26^

### Objective 4: self-identification of errors

The data around how providers, identified using an automated search method, catch their own errors is sparse as papers concentrated on the reasons for drug discontinuation, rather than error identification.^22^^,^^26^^,^^41^^,^^46^^,^^48^^,^^54^^,^^55^

Koppel et al^46^ states that errors were identified by other healthcare professionals, but also that physicians frequently reported they caught their own errors. However, the paper does not provide any information about how they caught them.

Levin et al^48^ produced a survey regarding WPOE that included a question regarding how they are caught. However, very few respondents could postulate how errors were identified before reaching the patient; only that some believe they are caught by the ordering physician.

The physicians interviewed stated errors were usually seen when reviewing the order after signing, with some errors intercepted by nurses. Often the prompt for nurses to realize an order was incorrect was when it did not correspond with the patient’s condition, or when a task they know needed to be completed did not appear in their task list. Less frequently, the error was spotted by a pharmacist.

Lombardi et al^54^ did not interview the clinicians involved in the study specifically, however clinicians reported anecdotally that the intervention (patient ID re-entry function) made them aware of an incorrect patient selection before the alert itself was triggered.

Hickman et al^55^ received 312 email responses from clinicians providing further information about the identified errors. However, they only reported the reasons for the discontinued orders (eg, wrong-patient, wrong-dose, etc.) rather than the identification method.

Abraham et al^41^ conducted 101 telephone interviews with clinicians involved in voided orders, with one question asking how the clinician identified the order needed to be voided. Unfortunately, the paper did not detail how providers identified their own errors. In contrast to Koppel et al,^46^ this paper describes the positive surveillance role of pharmacists in intercepting ordering errors. It stated that nearly half of the medication ordering errors identified were voided by pharmacists, but again, does not state how the errors were identified.

A more recent study by Abraham et al^26^ delved more deeply into the risk factors associated with medication ordering errors. They detail strategies that clinicians have developed to mitigate the risk of WPOE, such as reading the patient’s first and last name, double-checking the chart at the time of ordering, and reviewing the order entry before signing.

## DISCUSSION

MEs cost the healthcare sector large sums of money, and are responsible for thousands of deaths every year.^3–5^ Accounting for 21% of MEs, prescribing error rates have remained static for the last 20 years despite the increased prevalence of CPOE. WPOE errors are reported with increasing use of CPOE, causing potentially thousands of extra errors per year. Error investigations are commonly retrospective and use low quality data. This review identified a number of different automated search methods to prospectively identify WPOE errors.

### Error detection methods

As shown in Table 2, the RAR method of Adelman et al^22^ is by far the most commonly reported, used in a number of different studies. One advantage is that it is specific for WPOE. It also achieves a PPV of 76.2% (95% CI 70.6%–81.9%). Another study, albeit not identified by our search, reported a PPV for WPOE of 66.7% (95% CI, 54.7%–78.5%).^24^ These PPVs are comparable to the Void Alert Tool (VAT) of Abraham et al^26^^,^^41^ who report PPVs of 78 ± 1.2% and 93.1% (95% CI 88.1%–98.1%) for medication ordering errors.

It is a simple method which has been successfully used in a number of different systems, as well as coupled with a variety of electronic interventions.^23^^,^^49^^,^^53^^,^^54^

False positives were identified by Adelman et al^22^ and were factored into the PPV through the use of semistructured phone interviews with the providers. These false positive RAR events were often associated with rapidly discontinued orders placed on specialist rounds, such as “total parenteral nutrition (TPN) rounds,” and are not WPOE.^22^

Currently, the RAR method only detects WPOE. For the detection of other errors, such as wrong-drug or wrong-dose, further design and validation work is required.

The second most popular method is the VAT of Abraham et al.^41^ The method of Hickman et al^55^ uses the same mechanism; an alert based off the medication discontinuation reason selected. This alert includes a number of reasons for voided prescriptions; it is not specific for WPOE.

The VAT has reported PPVs of 78%^26^ and 93.1%^41^ for medication ordering errors, and 70.2%^41^ and 80.3%^26^ for accuracy of voiding reason, which is comparable to the PPV of the RAR method.^22^ The 2 VAT papers^26^^,^^41^ do not provide a PPV for WPOE, however their predecessor paper by Kannampallil et al^50^ reported a PPV of only 48 ± 10% for the accuracy of “wrong patient” as the voiding reason.

The VAT appears the easiest search method to use, but requires the prescribing system to have the appropriate medication discontinuation reasons. Unlike the RAR method, the detection of other errors would be easy to perform in the presence of the appropriate reasons offered to the provider, however the accuracy of the discontinuation reason chosen can vary considerably.

It should be noted there is a subtle difference between the RAR method and the VAT regarding what exactly is being detected; the VAT focuses on voided orders, whereas the RAR method looks at all discontinued and represcribed orders. While either discontinuing or voiding an order essentially does the same thing, selecting the void option indicates the order was originally placed in error. Some EHRs have both the void and discontinue options available for clinicians to choose from. In this way, the voiding option essentially acts as a voluntary error reporting system as the clinician is actively indicating the order was placed in error. Thus, if a user does not wish to admit to an error, they can simply discontinue it. As a result, there is the potential for a reporting bias that may affect the VAT results, as well as a potential bias in the qualitative results as the researchers would only be able to interview providers who actively chose to void an order.

### Error data

The data captured by the RAR method show good reproducibility across different studies and populations, demonstrating a general WPOE rate in the region of 31.8–60.6 per 100 000 orders.^22^^,^^43^^,^^45^^,^^51^ Higher rates of 142–202 per 100 000 orders were seen in Emergency Department-focused studies,^23^^,^^53^ which correlates with previously published literature. A low rate of 27.8 per 100 000 orders was reported in an intensive care setting, which could be explained by fewer patients and a slower patient turnover.^52^

Although fewer studies used the VAT, the general error rate was similar between Kannampallil et al^50^ and Hickman et al,^55^ with 490 “voids” per 100 000 orders and 450 “erroneous entries” per 100 000 orders, respectively. However, the Abraham et al^41^ study shows only 210 “voids” per 100 000 orders. This study was smaller, and pediatrics were excluded, but otherwise the difference has not been explained.

As reported above, the error rate detected by the VAT is around 10 times higher than when using the RAR method. This could be because the VAT is detecting all MEs that have been voided, whereas the RAR method is looking at WPOE specifically.

### Strategies to minimize errors

Multiple risk factors for WPOE have been identified, which would require a comprehensive strategy facilitating improvements across multiple levels to try and solve these issues. This review has managed to identify several interventions that effectively minimize errors.

Most interventions focused on interrupting the prescribing process, usually by presenting additional patient information, to encourage the prescriber to check they were prescribing for the correct patient. Examples include a patient ID-verification alert,^22^^,^^23^ a patient ID-reentry function,^22^^,^^54^ an indication-based CDS alert,^49^ and the continual display of patient photographs within the EHR.^23^^,^^53^

A method to reduce the risk of WPOE in newborns specifically was the development of a distinct first naming system at birth.^43^

Another solution, albeit one requiring an organizational culture change, would be to try and reduce the number of interruptions to clinicians currently performing a task.^22^

### Self-identification of error

The presence of WPOE data identified using the various automated search methods show that WPOEs are detected, however, there are gaps in the evidence as to how this occurs in practice. Multiple studies report that the errors are caught by other healthcare professionals, in addition to the original providers, but the processes and attributes of this remain unclear.

The strategies used by providers to identify their own errors have not been investigated thoroughly. Lombardi et al^54^ reported that a new patient-ID verification alert helped to make providers more aware of WPOE, but this was not a deliberate technique for avoiding WPOE, rather the raised awareness was a secondary effect from introducing the alert.

The only study that investigated the strategies used by providers to identify their own errors was Abraham et al.^26^ The strategies listed by the clinicians to reduce the risk of medication ordering is very useful, and suggests that a conversation about the patient, or with them, can help identify WPOE. This data would be most effective when incorporated into departmental mandatory training, enabling all practitioners to use these strategies to make their clinical practice safer.

Ultimately, further work based on a greater understanding of providers’ strategies is needed, to identify system changes that might lower the rate of WPOE.

#### Areas for improvement

Currently, the literature is dominated by only a small handful of researchers who often publish together. For the 15 papers included in this review, there are author credits assigned to 85 individuals. However, there is a large number of papers who share coauthors, with 21 of these authors listed on at least 2 papers. The most prolific author is credited on 5, which is one-third of the current literature. While this interrelatedness does not diminish the value of the work, it does suggest a need to broaden the community of scholars working in this field.

Other areas that need further research are the human factors and system interactions that contribute to the self-identification of ordering errors, as most of the work in this area is from one paper only.

### Limitations

The lack of homogeneity in the studies reviewed, made comparison between studies difficult. Broadly speaking, the studies either looked at background error rates in isolation, or in the context of an intervention. The automated search methods used either captured all MEs, or WPOE specifically. All the studies were conducted in the United States, so while providing valuable data on the WPOE phenomenon, limits the generalizability to other countries that employ CPOE systems. The lack of data regarding providers identifying their own errors could be a result of search bias, as any papers that did not specifically relate to automated searching for errors were excluded from the review. The papers reviewed in this study describe only self-intercepted near misses, and while a good proxy for real errors due to sharing the same causal pathway, are not the same and should not be treated as such.

## CONCLUSION

Several automated methods have been developed to identify MEs, with the ability of some to focus on WPOE specifically. The most popular methods are the RAR method of Adelman et al^22^ and the VAT/Erroneous entry alerts of Abraham et al^41^ and Hickman et al.^55^

The RAR method is the better choice for identifying WPOE due to its ease of use, national validation, and ability to be easily paired to any electronic prescribing system. However, if an investigator wished to investigate other errors involved in rapidly discontinued orders (eg, wrong dose, route, etc.), then the VAT/Erroneous entry alert would be the better option due to its ability to easily capture that data, albeit with some limitations.

## Supplementary Material

ooad057_Supplementary_Data

## Data Availability

No new data were generated or analysed in support of this research.
