# Supplementary material for: Automated search methods for identifying wrong patient order entry—a scoping review
Source: JAMIA Open. 2023 Aug 2;6(3):ooad057. doi: 10.1093/jamiaopen/ooad057 (PMC10397536; doi:10.1093/jamiaopen/ooad057)
Supplement: ooad057_Supplementary_Data [file ooad057_supplementary_data.docx]

# **SUPPLEMENTARY MATERIAL**

## **Appendix S1: Search strategy (example)**

Embase <1974 to 2021 November 16>

| **#** | **Query** | **Results from**  **17 Nov 2021** |
| --- | --- | --- |
| 1 | "e prescri*".mp. [mp=title, abstract, heading word, drug trade name, original title, device manufacturer, drug manufacturer, device trade name, keyword heading word, floating subheading word, candidate term word] | 975 |
| 2 | "electronic prescri*".mp. [mp=title, abstract, heading word, drug trade name, original title, device manufacturer, drug manufacturer, device trade name, keyword heading word, floating subheading word, candidate term word] | 4,230 |
| 3 | "computeri?ed prescription*".mp. [mp=title, abstract, heading word, drug trade name, original title, device manufacturer, drug manufacturer, device trade name, keyword heading word, floating subheading word, candidate term word] | 306 |
| 4 | "computeri?ed prescrib*".mp. [mp=title, abstract, heading word, drug trade name, original title, device manufacturer, drug manufacturer, device trade name, keyword heading word, floating subheading word, candidate term word] | 325 |
| 5 | "electronic order*".mp. [mp=title, abstract, heading word, drug trade name, original title, device manufacturer, drug manufacturer, device trade name, keyword heading word, floating subheading word, candidate term word] | 798 |
| 6 | "CPOE".mp. [mp=title, abstract, heading word, drug trade name, original title, device manufacturer, drug manufacturer, device trade name, keyword heading word, floating subheading word, candidate term word] | 1,761 |
| 7 | "computeri?ed physician order entry".mp. [mp=title, abstract, heading word, drug trade name, original title, device manufacturer, drug manufacturer, device trade name, keyword heading word, floating subheading word, candidate term word] | 1,468 |
| 8 | "computeri?ed prescriber order entry".mp. [mp=title, abstract, heading word, drug trade name, original title, device manufacturer, drug manufacturer, device trade name, keyword heading word, floating subheading word, candidate term word] | 188 |
| 9 | exp *computerized provider order entry/ | 1,993 |
| 10 | exp *physician order entry system/ | 139 |
| 11 | exp *electronic prescribing/ | 1,365 |
| 12 | "near miss*".mp. [mp=title, abstract, heading word, drug trade name, original title, device manufacturer, drug manufacturer, device trade name, keyword heading word, floating subheading word, candidate term word] | 4,023 |
| 13 | "patient safety incident*".mp. [mp=title, abstract, heading word, drug trade name, original title, device manufacturer, drug manufacturer, device trade name, keyword heading word, floating subheading word, candidate term word] | 704 |
| 14 | "medic* error*".mp. [mp=title, abstract, heading word, drug trade name, original title, device manufacturer, drug manufacturer, device trade name, keyword heading word, floating subheading word, candidate term word] | 43,504 |
| 15 | "medic* safety".mp. [mp=title, abstract, heading word, drug trade name, original title, device manufacturer, drug manufacturer, device trade name, keyword heading word, floating subheading word, candidate term word] | 5,423 |
| 16 | "error rate*".mp. [mp=title, abstract, heading word, drug trade name, original title, device manufacturer, drug manufacturer, device trade name, keyword heading word, floating subheading word, candidate term word] | 19,311 |
| 17 | (error* adj5 detect*).mp. [mp=title, abstract, heading word, drug trade name, original title, device manufacturer, drug manufacturer, device trade name, keyword heading word, floating subheading word, candidate term word] | 10,242 |
| 18 | "data analysis".mp. [mp=title, abstract, heading word, drug trade name, original title, device manufacturer, drug manufacturer, device trade name, keyword heading word, floating subheading word, candidate term word] | 290,617 |
| 19 | "quality improvement*".mp. [mp=title, abstract, heading word, drug trade name, original title, device manufacturer, drug manufacturer, device trade name, keyword heading word, floating subheading word, candidate term word] | 71,707 |
| 20 | "patient safety report*".mp. [mp=title, abstract, heading word, drug trade name, original title, device manufacturer, drug manufacturer, device trade name, keyword heading word, floating subheading word, candidate term word] | 231 |
| 21 | "error report*".mp. [mp=title, abstract, heading word, drug trade name, original title, device manufacturer, drug manufacturer, device trade name, keyword heading word, floating subheading word, candidate term word] | 1,517 |
| 22 | "prescri* error*".mp. [mp=title, abstract, heading word, drug trade name, original title, device manufacturer, drug manufacturer, device trade name, keyword heading word, floating subheading word, candidate term word] | 2,414 |
| 23 | "administration error*".mp. [mp=title, abstract, heading word, drug trade name, original title, device manufacturer, drug manufacturer, device trade name, keyword heading word, floating subheading word, candidate term word] | 1,358 |
| 24 | "dispensing error*".mp. [mp=title, abstract, heading word, drug trade name, original title, device manufacturer, drug manufacturer, device trade name, keyword heading word, floating subheading word, candidate term word] | 590 |
| 25 | "error type*".mp. [mp=title, abstract, heading word, drug trade name, original title, device manufacturer, drug manufacturer, device trade name, keyword heading word, floating subheading word, candidate term word] | 1,363 |
| 26 | [surveillance.mp](http://surveillance.mp/). [mp=title, abstract, heading word, drug trade name, original title, device manufacturer, drug manufacturer, device trade name, keyword heading word, floating subheading word, candidate term word] | 329,109 |
| 27 | "error detection".mp. [mp=title, abstract, heading word, drug trade name, original title, device manufacturer, drug manufacturer, device trade name, keyword heading word, floating subheading word, candidate term word] | 1,817 |
| 28 | ("wrong form*" or "wrong dos* form*" or "form* error*").mp. [mp=title, abstract, heading word, drug trade name, original title, device manufacturer, drug manufacturer, device trade name, keyword heading word, floating subheading word, candidate term word] | 423 |
| 29 | ("incorrect form*" or "incorrect dos* form*" or "form* error*").mp. [mp=title, abstract, heading word, drug trade name, original title, device manufacturer, drug manufacturer, device trade name, keyword heading word, floating subheading word, candidate term word] | 442 |
| 30 | ("wrong dos*" or "wrong dos* administration*" or "dos* error*").mp. [mp=title, abstract, heading word, drug trade name, original title, device manufacturer, drug manufacturer, device trade name, keyword heading word, floating subheading word, candidate term word] | 3,094 |
| 31 | ("incorrect dos*" or "incorrect dos* administration*" or "dos* error*").mp. [mp=title, abstract, heading word, drug trade name, original title, device manufacturer, drug manufacturer, device trade name, keyword heading word, floating subheading word, candidate term word] | 3,302 |
| 32 | ("wrong frequenc*" or "wrong dos* frequenc*" or "frequenc* error*").mp. [mp=title, abstract, heading word, drug trade name, original title, device manufacturer, drug manufacturer, device trade name, keyword heading word, floating subheading word, candidate term word] | 245 |
| 33 | ("incorrect frequenc*" or "incorrect dos* frequenc*" or "frequenc* error*").mp. [mp=title, abstract, heading word, drug trade name, original title, device manufacturer, drug manufacturer, device trade name, keyword heading word, floating subheading word, candidate term word] | 235 |
| 34 | ("wrong route*" or "wrong dos* route*" or "route error*").mp. [mp=title, abstract, heading word, drug trade name, original title, device manufacturer, drug manufacturer, device trade name, keyword heading word, floating subheading word, candidate term word] | 173 |
| 35 | ("incorrect route*" or "incorrect dos* route*" or "route error*").mp. [mp=title, abstract, heading word, drug trade name, original title, device manufacturer, drug manufacturer, device trade name, keyword heading word, floating subheading word, candidate term word] | 117 |
| 36 | "wrong patient*".mp. [mp=title, abstract, heading word, drug trade name, original title, device manufacturer, drug manufacturer, device trade name, keyword heading word, floating subheading word, candidate term word] | 584 |
| 37 | "incorrect patient*".mp. [mp=title, abstract, heading word, drug trade name, original title, device manufacturer, drug manufacturer, device trade name, keyword heading word, floating subheading word, candidate term word] | 227 |
| 38 | "wrong patient* identif*".mp. [mp=title, abstract, heading word, drug trade name, original title, device manufacturer, drug manufacturer, device trade name, keyword heading word, floating subheading word, candidate term word] | 13 |
| 39 | "incorrect patient* identif*".mp. [mp=title, abstract, heading word, drug trade name, original title, device manufacturer, drug manufacturer, device trade name, keyword heading word, floating subheading word, candidate term word] | 30 |
| 40 | exp *"near miss (health care)"/ | 102 |
| 41 | exp *patient safety/ | 21,615 |
| 42 | exp *adverse event/ | 210,118 |
| 43 | exp *medical error/ | 31,743 |
| 44 | exp *medication error/ | 9,779 |
| 45 | exp *drug dosage form/ | 131,833 |
| 46 | exp *patient identification/ | 1,260 |
| 47 | "retract and reorder".mp. [mp=title, abstract, heading word, drug trade name, original title, device manufacturer, drug manufacturer, device trade name, keyword heading word, floating subheading word, candidate term word] | 19 |
| 48 | "automat* detect*".mp. [mp=title, abstract, heading word, drug trade name, original title, device manufacturer, drug manufacturer, device trade name, keyword heading word, floating subheading word, candidate term word] | 9,906 |
| 49 | detect*.mp. [mp=title, abstract, heading word, drug trade name, original title, device manufacturer, drug manufacturer, device trade name, keyword heading word, floating subheading word, candidate term word] | 3,344,259 |
| 50 | 1 or 2 or 3 or 4 or 5 or 6 or 7 or 8 | 7,857 |
| 51 | limit 50 to abstracts | 6,512 |
| 52 | 9 or 10 or 11 | 2,122 |
| 53 | 51 or 52 | 7,071 |
| 54 | 12 or 13 or 14 or 15 or 16 or 17 or 18 or 19 or 20 or 21 or 22 or 23 or 24 or 25 or 26 or 27 or 28 or 29 or 30 or 31 or 32 or 33 or 34 or 35 or 36 or 37 or 38 or 39 or 47 or 48 or 49 | 3,981,915 |
| 55 | limit 54 to abstracts | 3,842,304 |
| 56 | 40 or 41 or 42 or 43 or 44 or 45 or 46 | 393,679 |
| 57 | 55 or 56 | 4,193,265 |
| 58 | 53 and 57 | 2,876 |
| 59 | limit 58 to english language | 2,724 |

## **Appendix S2: Themes**

Themes produced by Abraham et al,.([35](#_ENREF_35))

| **Contributing Factors** | |
| --- | --- |
| Technology | - Inadequate alert design - Ineffective CPOE interface design - Inability to view all active medication lists on single page - Limited CPOE experience - Limited interoperability between eMAR and CPOE - Multiple opened charts - Other (please explain) |
| Cognitive | - Alert fatigue - Multitasking - Misinterpretation of verbal order - Dose calculation mistake, - Decimal point inaccuracy - Similar patient names - Confusing patient MRNs - Negligence - Fatigue - Mix-up of patient charts - Other (please explain) |
| Social | - Limited communication between care team - Lack of order notification - Limited knowledge of drugs - Incorrect drug formulation/strength - Rule violations - Limited protocol awareness - Memory lapses - Other (please explain) |
| Environmental | - Noise Lighting issues - Distractions - Interruptions - Incorrect equipment programming - Other (please explain) |
| Organizational | - High clinician workload - Staffing issues - Error reporting culture - Other (please explain) |
